# Medical pattern classification using a novel binary similarity approach based on an associative classifier

**DOI:** 10.3389/frai.2025.1610856

**Published:** 2026-01-14

**Authors:** Osvaldo Velazquez-Gonzalez, Antonio Alarcón-Paredes, Cornelio Yañez-Marquez

**Affiliations:** Centro de Investigación en Computación, Instituto Politécnico Nacional, Mexico City, México

**Keywords:** binary similarity, classification algorithms, machine learning, medicine dataset, pattern classification, pattern recognition

## Abstract

Classification is a central task in machine learning, underpinning applications in domains such as finance, medicine, engineering, information technology, and biology. However, machine learning pattern classification can become a complex or even inexplicable task for current robust models due to the complexity of objective datasets, which is why there is a strong interest in achieving high classification performance. On the other hand, in particular cases, there is a need to achieve such performance while maintaining a certain level of explainability in the operation and decisions of classification algorithms, which can become complex. For this reason, an algorithm is proposed that is robust, simple, highly explainable, and applicable to datasets primarily in medicine with complex class imbalance. The main contribution of this research is a novel machine learning classification algorithm based on binary string similarity that is competitive, simple, interpretable, and transparent, as it is clear why a pattern is classified into a given class. Therefore, a comparative study of the performance of the best-known state-of-the-art classification algorithms and the proposed model is presented. The experimental results demonstrate the benefits of the proposal in this research work, which were validated through statistical hypothesis tests to assess significant performance differences.

## 1 Introduction

This study proposes a novel algorithm for pattern classification. The proposed algorithm is primarily based on binary string similarity and is called the N-Similarity Binary Classifier (n-SBC), as it uses the Hamming string similarity method and a binary-value encoder called the reflected binary code (RBC) or Gray code. The creation, design, implementation, and application of n-SBC support the solution-finding process for the problem represented by the supervised case in pattern classification.

Humans can recognize objects, actions, and everyday elements (patterns); however, what is simple for humans can be a very complex problem for a computational algorithm. The discipline that includes in its field of study the modeling and programming of automatic object and action recognition tasks is Pattern Recognition (PR) (Sarker, 2021). There are four basic tasks of PR: classification, regression, recovery, and clustering (De Sa, 2012; Rane et al., 2024). The first three are located in the supervised learning paradigm, while the last one is the emblematic task of the unsupervised learning paradigm (Janani and Vijayarani, 2019). In this study, emphasis will be placed on the first task of the supervised learning paradigm: intelligent pattern classification, or machine learning for pattern classification. In the state of the art, a wide variety of conceptual bases provide theoretical support for the task, such as Bayesian classifiers and distance-based models like kNN. Based on decision trees (C4.5 or Random Forest), based on and inspired by the neurons of the human brain (Multilayer Perceptron), or based on optimization of analytical functions, such as support vector machines (Bhargavi and Jyothi, 2009; Cover and Hart, 1967; McCulloch and Pitts, 1943; Quinlan, 1990; Rosenblatt, 1958; LeCun et al., 2015; Cortes, 1995). As important assistants in the development of PR and related disciplines, there are dataset repositories (Dua and Graff, 2019) and certain platforms where some research groups make available to users valuable computational tools, as well as implementations of algorithms and methods; such is the case of WEKA (Hall et al., 2009) and KEEL (Bhargavi and Jyothi, 2009; Cover and Hart, 1967; McCulloch and Pitts, 1943; Quinlan, 1990; Rosenblatt, 1958; LeCun et al., 2015; Cortes, 1995), two of the most useful, famous and popular platforms.

Before 1997, when the No Free Lunch Theorem (De Sa, 2012; Duda et al., 2001) was published, a large number of research groups were trying to find the best classifier; however, this theorem resulted in researchers concluding that this search is futile, since there is no intelligent pattern classifier that is the best in all cases. Therefore, researchers' efforts are currently directed toward finding alternatives to improve the performance of pattern classifiers, recognizing that there is no best one. One of the main recent achievements has been the development of a new pattern classification paradigm, Minimalist Machine Learning (MML) (Yáñez-Márquez, 2020). It is in this context that the central proposition of this work arises.

Recently, significant efforts have been devoted to finding alternatives to improve the performance of intelligent pattern classifiers, recognizing that there is no single best approach. In these research processes, a wide variety of tools and methodologies developed over the decades are used. Thus, one recurring theme in the generated algorithms is the use of associative models. These associative models are not designed for intelligent pattern classification but rather for pattern retrieval; however, if the designer adequately represents the output patterns, they can perform the classification task correctly. The first associative model recorded is the Lernmatrix, created in 1961 by Steinbuch (1961), followed by the associative model called the Correlograph, whose creation and publication occurred 8 years later (Willshaw et al., 1969). The year 1972 saw the birth of one of the best-known associative models: the Linear Associator, which emerged as the fusion of two independent models (Kohonen, 1972; Anderson, 1972); from then on, a considerable number of associative models have been generated in the world with successful applications in various areas of human activity (Hopfield, 1982; Talib, 2018; Ibrahim and Abdulazeez, 2021). It is pertinent to note that research on the subject is ongoing (Hoffmann, 2019; Nozari et al., 2024; Zhu et al., 2024; Bian and Priyadarshi, 2024).

Early detection of diseases has increased its relevance in recent years due to the various benefits that have a beneficial impact on public health, such as increasing the chances of survival in patients suffering from severe respiratory diseases (Vayadande, 2024; Rasool et al., 2023) and achieving a better recovery thanks to detection at an early stage of the disease. Research focused on pre-diagnosis of respiratory diseases has recently gained momentum worldwide, with widespread interest in improving early detection. Currently, both invasive and non-invasive methods are applied. However, lately, the use of machine learning classification algorithms for disease diagnosis has become an increasingly important area of research globally due to their ease of implementation and accessibility (Rana and Bhushan, 2023). This has caused frequent research in the literature on the development of novel specialized models for the medical pre-diagnosis of all types of diseases (Kumar et al., 2023; Ahsan et al., 2022).

In this paper, elements of associative models have been taken in order to create and design the main algorithm of the proposal, but in addition to these elements, the concept of string similarity has been used, as well as the Hamming distance and a binary pattern encoder, the reflected binary code (RBC or Gray code). The rest of this paper is organized as follows: Section 2 details the related works. Section 3 describes the novel proposal algorithm, with detailed examples of its operations in training and classification phases. In Section 4, the experimental phase and results are presented, and, finally, in Section 5, the conclusions and future research are included.

## 2 Related research

As discussed above, assuming the existence of a universally optimal pattern classification algorithm is unmotivated due to the no free lunch theorem, forcing researchers in machine learning-related areas to focus on improving the performance of existing models and thereby reducing classification error (Bui et al., 2020; Shehadeh et al., 2021; Misra and Yadav, 2020). Alternatively, some studies propose entirely novel machine learning models for pattern classification, with the aim of exploring new possibilities, as demonstrated by Amygdalos et al. (2023) and Hissou et al. (2023). Similarly, researchers have pioneered the development of new algorithms based on associative memories, including those by Moreno-Ibarra et al. (2021), (Yang and Ding 2020), and Luna-Ortiz et al. (2023).

Section 2 is divided into three parts. Section 2.1 describes the Hamming Distance algorithm, a fundamental concept for our novel pattern classification algorithm. Section 2.2 explores the RBC (Reflected Binary Code), another crucial element of our novel method for converting the original dataset into binary strings. Finally, Section 2.3 provides an overview of the state-of-the-art machine learning algorithms, including both well-known models and associative memories used for classification tasks, as well as a deep dive into current research on Hamming Distance and RBC in machine learning.

### 2.1 Hamming distance

The Hamming distance, the most used metric with binary strings and a natural similarity measure on binary codes, can be computed with just a few machine instructions per comparison (Pappalardo et al., 2009). The computational effort required to calculate the Hamming distance linearly depends on the size of the string, and it is often used to quantify the extent to which two bit-strings of the same dimension differ (Norouzi et al., 2012; Bookstein et al., 2002).

The distance is defined as the minimum number of errors that could transform a pattern *A* into a pattern *B*, i.e., it measures the minimum number of values that must be changed to transform a string into another target string (Zhang et al., 2013).

Another way to define it could be the number of positions at which the corresponding bits are different, that is, express it as the following (Gaitanis et al., 1993):


$$\begin{array}{l} D\left(A,B\right)=\overset{n}{\underset{i=1}{\sum }}|A_{i}-B_{i}|,\;A_{i},B_{i}\;\in \left\{0,1\right\}\;, \end{array}$$ (1)

where *A*_*i*_ and *B*_*i*_ are the bits at the *i*-th position of the respective strings. And the subtraction refers to the XOR logic gate operation. The use of the Hamming distance has many applications, the most relevant being in coding theory, the electronics field, and term clustering (Norouzi et al., 2012). It has been shown that one can perform exact nearest-neighbor search in Hamming space significantly faster than linear search, achieving sublinear run times.

### 2.2 Reflected Binary Code (RBC)

The Gray encoder, also known as Reflected Binary Code (RBC), was invented by Frank Gray in 1953 in a Bell Telephone Laboratories patent (Agrell et al., 2004; Doran, 2007; Goodall, 1951). It is a binary numbering system in which the main property is that two adjacent values differ by only a single digit. For example, value 2 differs from values 1 and 3 in RBC by a single digit. Table 1 is an illustrative example.

**Table 1:** Example of the single distance of the Gray binary code (RBC).

**Decimal**	**Binary code**	**RBC (gray code)**
1	001	001
2	010	011
3	011	010
4	100	110
5	101	111

In this case, unlike the classic binary encoder, the bit difference between an adjacent decimal value is only one digit. In this sense, this advantage helps preserve similarity between neighboring patterns, unlike standard binary encoding, which can cause adjacent values to differ across multiple bits, creating more complex relationships between close patterns. Therefore, this helps and supports the performance of our proposed classifier, as explained in Section 3, since it is based on string binary simultaneities.

This system binary code is commonly used to refer to any single distance. Its unique characteristics make it very useful across different domains, especially for error correction, position encoders, genetic algorithms, and digital communication (Agrell et al., 2004; Bhat and Savage, 1996).

To obtain a binary string using RBC, it can be done as follows: First, convert the decimal value to classic binary code, and subsequently convert from binary code to RBC, applying XOR (exclusive OR) to each bit with the right bit, excluding the most significant bit. For example, let us say we want to convert the number 5 into RBC. First, the binary value of 5 is 101, and the MSB in this case is 1. Now, applying the XOR operation, starting from the right to the left but going on the right side, taking the second bit (0) and applying XOR with the first bit (1), the result is 1; then taking the third bit (1) and applying XOR with the second bit (0), the result is 1; and finally, concatenating the MSB as the first bit of the resulting string after the XOR operations; therefore, the RBC of the number 5 is 111 (Bhat and Savage, 1996).

Before converting decimal values to binary strings using the RBC method, the dataset values are preprocessed: the minimum value per feature is computed; if required, the decimals are truncated to 2 decimal places; and finally, the values are rounded to integers. This aims to obtain only positive integer values.

To illustrate the conversion to integer values and truncation, the following example is provided. Consider a continuous numeric feature:


$$\begin{array}{l} \left\{1.131,\;-0.010,\;1.351,\;-0.110,\;0.660,\;1.411\right\} \end{array}$$ (2)

In this case, to obtain only positive numbers, the minimum value is the sum of all the values of the feature array, which in this case is −0.11, obtaining the following result:


$$\begin{array}{l} \left\{1.241,\;0.100,\;1.461,\;0.000,\;0.770,\;1.521\right\} \end{array}$$ (3)

Then, it is truncated to two decimals only:


$$\begin{array}{l} \left\{1.24,\;0.10,\;1.46,\;0.00,\;0.77,\;1.52\right\} \end{array}$$ (4)

Subsequently, all the values of the feature are escalated to integer values, such as


$$\begin{array}{l} \left\{124,\;10,\;146,\;0,\;77,\;152\right\} \end{array}$$ (5)

Finally, using these feature values, the RBC binary string is computed. Table 2 shows an example of how the binary codes look after RBC encoding.

**Table 2:** Illustration of RBC after preprocessing.

**Decimal**	**Binary code**	**RBC (gray code)**
124	01111100	01000010
20	00011000	00010100
146	10010010	11011011
0	00000000	00000000
77	01001101	01101011
152	10011000	11010100

### 2.3 Pattern classification algorithms

In the current state of the art, many machine learning algorithms focus on classification tasks. Some of them are based on distance, such as the kNN (k-nearest neighbors) model (Zhang, 2021), while others are based on optimization, such as SVM (support vector machines) (Abdullah and Abdulazeez, 2021). Others are based on decision trees (Costa and Pedreira, 2023), such as C4.5, or bagging approaches such as the random forest algorithm. In more recent literature, models are inspired by biological concepts, such as the human brain. For instance, the multilayer perceptron (an artificial neural network) falls into this category. Currently, the most widely used are deep learning models (Sharifani and Amini, 2023), which are neural networks with many layers and additional specialized preprocessing stages, such as CNNs (convolutional neural networks) for image processing and transformers and embedding approaches for natural language processing tasks (Galli et al., 2024).

#### 2.3.1 Associative memories

An associative memory ***M*** is a pattern-input/output system whose primary purpose is to learn to correctly retrieve complete patterns from inputs that may be corrupted by several sources of noise. Can be expressed as *x* → ***M*** → *y*. The input and output patterns are represented by the column vectors *x* and *y*, respectively. Each input is associated with a corresponding output pattern; such an association is expressed as (*x, y*). Memory ***M*** is represented by a matrix. This matrix is formed from a finite set of previously known associations, known as the fundamental set (considered as the learning stage).

Finally, the retrieval process (which could be known as the classification stage if the designer made adequate changes) consists of performing the memory *M* with the given steps for that phase, with the aim of finding enough conditions to obtain the fundamental output pattern *y* from the pattern *x* (Nozari et al., 2024).

In the state of the art, there are pioneers of associative memory with their original purpose as retrieval machines, such as Steinbuch's Lernmatrix and Linear Associator (Steinbuch, 1961; Nozari et al., 2024). In these models, learning is typically implemented by updating a memory matrix with a set of rules. For example, in a Lernmatrix, each association (*x, y*) contributes an update of the form *M* ← *M* + *yx*. During inference, the unknown pattern *x* is projected through the learned memory, *y* = *Mx*, and a non-linear function (e.g., thresholding) produces the retrieved output pattern. If class labels are encoded as output vectors (e.g., one-hot) and the decision is taken from *y* by a winner-take-all rule, the same associative mechanism can be used as a classifier. This idea is exploited in modern associative classifiers (Velazquez-Rodriguez et al., 2020), which extend the classical Lernmatrix with a novel mathematical transformation that makes the matrix updates and recall rule suitable for supervised pattern classification rather than only for pattern completion.

Associative memories are relevant because the proposed n-SBC classifier was inspired by them. In n-SBC, training patterns are stored as rows in a kind of memory matrix. Then, given a test or unknown pattern, similarity is computed as the bitwise overlap under bipolar coding, which amounts to an affine transformation of an inner product, unlike classic associative memories that learn from projections. Thus, our novel model is conceptually linked to associative memories but implements a different representation (RBC codes) and a Hamming-based decision rule tailored to pattern classification.

#### 2.3.2 Hamming distance and RCB in pattern classification

After an extensive documentary search, it was found that, throughout history, there have been very few attempts to create intelligent pattern classification algorithms based on the Hamming distance. Regarding the RBC code, no impactful work has been found; therefore, this proposal uses the Hamming distance and the RBC code simultaneously within the same pattern classifier algorithm. At the same time, the proposal's novelty and originality are ensured. The closest is a work using RBC codes, published in 2017 (Šarkovskis et al., 2017), the authors Šarkovskis, Jeršovs, Kolosovs and Grabs describe the functionality of a real-time classifier useful for the computation of statistical parameters of data streams, the detection of symbols of different modulation types and other applications where the fastest possible association of a sample of input signals with one of the predefined categories is required.

While explicit RBC and Hamming classifiers are rare in the literature, some frameworks and methodologies encode data into binary codes and perform comparisons, mainly in hashing and ECOC-style multiclass reduction. In the ECOC (error-correcting output codes) methodology, each class is assigned a binary codeword, and a bank of binary base learners (e.g., C4.5, SVM) is trained, one per code column, as described by Dietterich and Bakiri (1994). Then, to perform classification, the column outputs are concatenated, and the label is chosen as the nearest class codeword, typically via the Hamming distance. Thus, ECOC is an ensemble framework, not a classifier *per se*: it improves the underlying learners but does not replace them (Dietterich and Bakiri, 1994).

On the other hand, in learning-to-hash or supervised hashing, features are transformed into compact binary codes by a learned encoder, and classification is commonly implemented via Hamming space (e.g., *k-*NN or ranking codes); this establishes that distance is an effective similarity for large-scale prediction when inputs are binary encoded (Norouzi et al., 2012). Thus, supervised hashing is not a classifier *per se* but a representation-learning method whose downstream machine-learning models operate on the learned bits. This approach offers fast lookups once trained, but it introduces training complexity, and results depend on the codebook.

Our contribution to n-SBC differs from these strands in two quantitative ways. First, unlike supervised hashing, which learns codebooks and then delegates prediction to a classic classifier (e.g., *k-*NN), n-SBC uses a deterministic RBC mapping per feature and classifies by Hamming distance, removing the encoder learning stage while preserving fast bit-wise comparisons. In supervised hashing, performance depends on the learned encoder; in n-SBC, performance hinges on the RBC representation and Hamming aggregation. Second, whereas ECOC emphasizes maximizing inter-class Hamming separations between class codewords and requires training a bank of binary base learners (e.g., C4.5, SVM), thus acting as a framework rather than a classifier *per se*, n-SBC treats the entire RBC binary string instance as the object of comparison, performing instance Hamming matching rather than decoding to a fixed class codeword. In short, both models are enabling methods that rely on baseline models (C4.5, SVM, *k-*NN, etc.), whereas n-SBC is the classifier itself. Together with our operational unification of RBC and Hamming, these distinctions place n-SBC at a different point in the design space (Xiao et al., 2022). A summary of the main differences is shown in Table 3.

**Table 3:** Comparison between n-SBC and related research.

**Method**	**Representation**	**Decision rule**	**Distance metric**	**Explicability**	**Key distinction**
ECOC	Class codewords (binary) + any base classifier model	Nearest class codeword from trained base models	Hamming	Bits reflect learned columns, not original features	Framework to improve baseline classifiers with Hamming and encoders
Supervised hashing	Learned binary codes	k-NN, ranking in Hamming space	Hamming	Hash bits are opaque codes, so each feature is not accessible	Methodology to improve baseline classifiers with Hamming and encoders
Associative Memories	Learned binary codes	Linear projection	None	Shows which stored patterns are recalled but not feature-influenced	Classical associative retrieval
k-NN	Raw or normalized real values	k-NN in real space	Euclidean, Chebyshev, Manhattan, Minkowski	Show neighbors who influenced the decision	A classifier supporting different distance metrics
n-SBC	Deterministic RBC per feature, then full binary string	Instance-level Hamming over RBC encode	Hamming	Bit maps to feature segments and show similar patterns influenced by the decision	Classifier model using simple RBC and Hamming

To make these differences concrete, a compact comparison table covering code construction and decision rule, along with a small ablation replacing RBC with a fixed-width standard binary encoding to isolate RBC's contribution. As discussed, RBC improves n-SBC because adjacent codes differ by only one bit, preserving similarity between neighboring values; in contrast, standard binary encodings may flip multiple bits between consecutive values, distorting local neighborhoods and weakening bit-wise interpretability.

Therefore, unlike ECOC and supervised hashing, and unlike classical *k-*NN in feature space, n-SBC is an associative classifier whose decision rule operates directly in Hamming space.

## 3 Our proposal model

In this section, the main idea of the N-Similarity Binary Classifier (n-SBC) algorithm is explained, along with its operation; the learning phase and, finally, the classification phase of the proposed algorithm are addressed. The proposed algorithm is primarily based on the Hamming string similarity method and the reflected binary code (RBC) encoder, also known as the Gray Code, both of which are fundamental components of the model. The purpose of this study is to improve the performance of associative approach classifiers across several medical datasets to enhance disease detection.

To address the issue of missing values and categorical data, our proposed method requires preprocessing the dataset to address this complexity beforehand. To handle missing values, the classic imputation method was applied, replacing missing values with the mean for numerical data and the mode for categorical data. This resulted in datasets without missing values when present. Finally, the categorical variables were converted using the classic label encoding method, which assigns each category a unique numeric value.

Then, the RBC method is applied to the entire dataset. In this case, every feature of the input patterns *x*^μ^ is converted to their equivalent binary RBC code to obtain a p-dimensional binary string, where p represents the maximum length of the largest converted value, denoted as $b_{i}^{\mu }=RBC\left(x_{i}^{\mu }\right)$.

In order to obtain a single binary string, we concatenate each transformed feature together, expressed as follows:


$$\begin{array}{l} b^{\mu }=\left(b_{1}^{\mu },b_{2}^{\mu },\ldots ,b_{i}^{\mu }\right) \end{array}$$ (6)

Let us assume that there is a dataset *D*, divided into two subsets: *L* and *T*, for learning and testing, respectively.

### 3.1 Learning phase for the proposed approach

The learning phase of the n-SBC model has only one step. It consists of creating a memory matrix, denoted by *M*, which contains every transposed binary string pattern of the learning dataset *L*, generated previously by applying the RBC code to each pattern. Finally, on the matrix *M*, each element corresponds to the entire binary string representation of *b*^μ^, expressed as follows:


$$\begin{array}{l} M=\{\begin{array}{c} {b^{1}}^{T}\\ {b^{2}}^{T}\\ \vdots \\ {b^{L}}^{T} \end{array}\} \end{array}$$ (7)

Algorithm 1 **Training of n-SBC with RBC coding.**

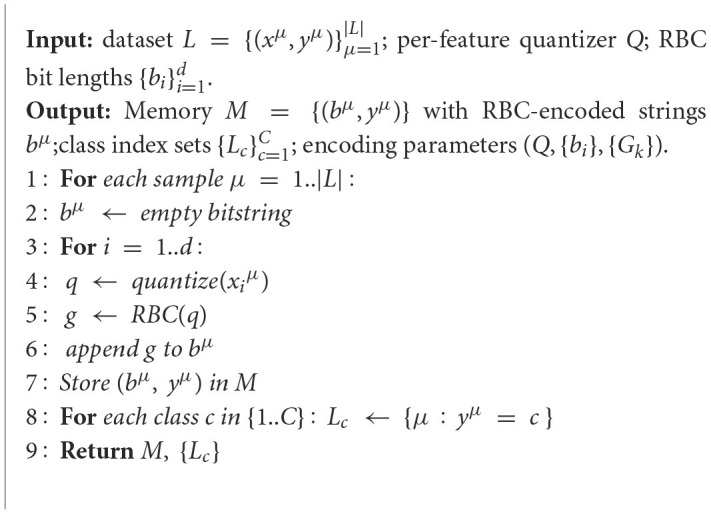

### 3.2 Classification phase for the proposed approach

The classification phase of the n-SBC model has four stages; the first is the calculation of the Hamming Distance between the unknown pattern *x*^ω^ to each pattern of the dataset *L*. To calculate it, first let us assume that the unknown pattern has already undergone the RBC transformation, yielding *b*^ω^. Therefore, the Hamming distance, *H*(*b*^ω^, *b*^μ^), represents the number of positions at which the corresponding bits are different. The above is expressed as follows:


$$\begin{array}{l} H\left(b^{\omega },b^{\mu }\right)=\overset{u}{\underset{j=1}{\sum }}|b_{j}^{\omega }-b_{j}^{\mu }|, \end{array}$$ (8)

where *u* is the dimensionality of the patterns. $b_{j}^{\omega }\;and\;b_{j}^{\mu }$ represent the *j*-th elements of the pattern *b*^ω^ and the training dataset pattern *b*^μ^.

This first step resulted in a vector distance, denoted as *Z*, which contains the result of the subtraction of the cardinality per dataset, denoted as *u*, with the computed Hamming distance of each pattern for the dataset *L* to the interested pattern *b*^ω^. The dimension of *Z* is equivalent to the cardinality of the dataset *L*, we can represent it with the following expression:


$$\begin{array}{l} Z^{\omega }=(\begin{array}{c} u-H\left(b^{\omega },b^{1}\right)\\ u-H\left(b^{\omega },b^{2}\right)\\ \vdots \\ u-H\left(b^{\omega },b^{\mu }\right) \end{array}) \end{array}$$ (9)

The second stage of the classification phase consists of handling the generated vector *Z*^ω^ to determine the class.

First, let *C* be the set of all the classes, such that: *C* = {*k*_1_, *k*_2_, …, *k*_*c*_}, where *c* is the number of classes. Then, let us introduce *K*_*i*_ to denote the number of patterns present within the *i*-th class, expressed as *K*_*i*_ = |*k*_*i*_|, ∀*i* ∈ {1, …, *c*}. Now, we determine the smallest pattern count across all the classes, termed *K*_min_, such as follows:


$$\begin{array}{l} K_{\min}=(K_{i}) \end{array}$$ (10)

Subsequently, for any integer *n* satisfying 1 ≤ *n* ≤ *K*_min_, we extract the *n*-th largest component from the vector *Z*^ω^ of each class, represented as $Z_{i}^{n}$. The hyperparameter *n* controls how many of the largest components are aggregated, and different values of *n* correspond to different versions of the classifier. Finally, a vector *y*^*n*^ is created by applying a sum to the selected *n*-th largest components, therefore, *y*^*n*^ is calculated by the following expression:


$$\begin{array}{l} y^{n}=(\begin{array}{c} \sum_{i=1}^{s}Z_{\left(i\right)}^{1}\\ \sum_{i=1}^{s}Z_{\left(i\right)}^{2}\\ \vdots \\ \sum_{i=1}^{s}Z_{\left(i\right)}^{j} \end{array}), \end{array}$$ (11)

where in this case *s* represents the number of samples of each *i*-class in the dataset. The third step consists of assigning to the unknown pattern *x*^ω^ his corresponding class *y*^ω^. For that, we update the vector *y*^*n*^ with the following rule:


$$\begin{array}{l} y_{i}^{\omega }=\{\begin{array}{c} 1\;if\;y_{i}^{n}\geq \vee_{j=1}^{p}y_{j}^{n}\\ 0\;otherwise \end{array} \end{array}$$ (12)

Finally, the fourth stage consists of calculating the predicted class of the unknown pattern *x*^ω^ using the one-hot vector created in stage three. Therefore, the class is assigned based on the position of the hot value, which indicates the predicted class, because each row of the vector corresponds to a class in the dataset. Meeting the following expression $y^{\omega }=\overset{C}{\underset{i=\;1}{\sum }}i^{*}y_{i}^{\omega }$.

One of the advantages of the n-SBC is that it aggregates only the top similar *n* components per class, so additional majority of samples do not grow a class's evidence unboundedly. Besides, the RBC encoding preserves similarity structure (adjacent numeric values differ by one bit), so compact minority clusters remain coherent in Hamming space and can dominate the selected top *n* samples. Consequently, decisions are driven by local match quality rather than by class prevalence, thereby mitigating the typical bias toward the majority class. This can enhance model performance with imbalanced complexity data.

Regarding the scope of applicability, n-SBC tends to perform well when classes exhibit locally coherent neighborhoods in feature space and when similarity is meaningfully captured by RBC. It may underperform when features are highly non-monotonic or noisy, when classes strongly overlap, or when *B* is inflated by many irrelevant bits.

Algorithm 2 **Classification of n-SBC.**

**Input:** Query *x*^ω^; memory *M*; encoding parameters (*Q*, {*b*_*i*_}, {*G*_*k*_}); top-*n* policy (global *n*or per-class {*n*_*c*_}).
**Output:** Predicted class *y*.
1 : *b*^ω^ ← *encode x*^ω^
2 : **For** *each class c in* {1..*C*} :
3 : **For** *k* = 1..*K* :
4 : *s*_*c*_, *k* ← 0
5 : **For** *each μ in L*_*c*_ :
6 : **For** *k* = 1..*K* :
7 : *u* ← *bits of b*^ω^ *in G_k_*
8 : *v* ← *bits of bˆμ in G*_*k*_
9 : *s*_*c*_, *k* ← *s*_*c*_, *k* + ( |*G*_*k*_| − *Haming* (*u, v*) )
10 : *T*_*c*_ ← *indices of the n* (*or n*_*c*_) *largest values in* {*s*_*c*_, 1.. *s*_*c*_, *K*}
11 : *S*_*c*_ ← *sum* < *uscore* > {*k* ∈ *T*_*c*_} *s*_*c*_, *k*
12 : *y* ← *argmax*_*c*_ *S*_*c*_
13 : **Return** *y, along with* {*T*_*c*_} *for explanation*

### 3.3 Example of the train and classification phase for n-SBC

Below, a simplified example of the operation process of the learning and classification phases of our proposed classifier, the n-SBC model, is presented in detail. The patterns used for this practical example are detailed, where *x*^1^ and *x*^2^ belong to class *A*, while patterns *x*^3^, *x*^4^ and *x*^5^ belong to class *B*.


$$\begin{array}{l} x^{1}=\left(\frac{0.28}{0.17}\right);\;x^{2}=\left(\frac{0.21}{0.09}\right)\;x^{3}=\left(\frac{0.06}{-0.15}\right);\;x^{4}=\left(\frac{-0.24}{0.01}\right);\\ \;x^{5}=\left(\frac{0.07}{-0.28}\right) \end{array}$$ (13)

After applying the reflected binary code (RBC) and in order to maintain a column vector when concatenating the binary strings obtained, the following patterns result:


$$\begin{array}{r} b^{1}=RBC\left(x^{1}\right)=\left(\begin{array}{c} 1\\ 0\\ 1\\ 1\\ 1\\ 1 \end{array}\right);\;b^{2}=RBC\left(x^{2}\right)=\left(\begin{array}{c} 1\\ 1\\ 1\\ 1\\ 1\\ 0 \end{array}\right);\;\\ b^{3}=RBC\left(x^{3}\right)=\left(\frac{\begin{array}{c} 0\\ 1\\ 0 \end{array}}{\begin{array}{l} 0\\ 0\\ 1 \end{array}}\right);\\ b^{4}=RBC\left(x^{4}\right)=\left(\begin{array}{c} 0\\ 0\\ 0\\ 0\\ 1\\ 0 \end{array}\right);\;b^{5}=RBC\left(x^{5}\right)=\left(\begin{array}{c} 1\\ 1\\ 0\\ 0\\ 0\\ 0 \end{array}\right) \end{array}$$ (14)

Following the Equation 2, the matrix *M* is created, which contains every transposed binary string representation pattern that will be handled in the classification phase, in this case, is expressed as follows:


$$\begin{array}{l} M=\left(\begin{array}{c} {b^{1}}^{T}\\ {b^{2}}^{T}\\ {b^{3}}^{T}\\ {b^{4}}^{T}\\ {b^{5}}^{T} \end{array}\right) \end{array}$$ (15)

At this point, the learning phase is complete. We have all the binary strings from the learning dataset ready to manipulate and proceed with the following steps for inference. Then, for the classification phase, the vector *Z*^ω^ is created based on the Hamming distances of each pattern and the unknown pattern *x*^ω^. However, before obtaining the distance matrix, we define *x*^ω^ as follows:


$$\begin{array}{l} x^{\omega }=\left(\frac{0.16}{0.05}\right)\to b^{\omega }=\left(\begin{array}{c} 1\\ 1\\ 0\\ 0\\ 1\\ 0 \end{array}\right) \end{array}$$ (16)

Therefore, the *Z*^ω^ is denoted as follows. In this case, *u* = 6 because the dimensionality of each pattern is six.


$$Z^{\omega }=\left(\begin{array}{l} u-H\left(b^{\omega },b^{1}\right)=6-4\\ u-H\left(b^{\omega },b^{2}\right)=6-2\\ u-H\left(b^{\omega },b^{3}\right)=6-3\\ u-H\left(b^{\omega },b^{4}\right)=6-2\\ u-H\left(b^{\omega },b^{5}\right)=6-1 \end{array}\right)=\left(\begin{array}{l} 2\\ 4\\ 3\\ 4\\ 5 \end{array}\right)$$ (17)

At this stage, we must define the value of *n*, which in this example we define as *n* = 2. Having established the necessary parameters, we instantiate the vector *y*^*n*^ following Equation 11.


$$\begin{array}{l} y^{n}=\left(\begin{array}{c} 4\;+\;2\;=\;6\\ 5\;+\;4\;=\;9 \end{array}\right), \end{array}$$ (18)

where the *n* largest components for each class are summed to create the column vector *y*^*n*^. These components correspond to positions 1 and 2 in class A, and to positions 3, 4, and 5 in class B. Therefore, since the components with a higher *Z*^ω^ vector, which means they have greater similarity to the unknown pattern, suggest that they are similar to those samples that belong to class B. This information can be used to clarify the model's explainability. Finally, based on the rule defined previously in Equation 12, we update the vector *y*^*n*^ obtaining *y*^ω^.


$$\begin{array}{l} y^{\omega }=\left(\begin{array}{c} 0\\ 1 \end{array}\right) \end{array}$$ (19)

In this example, due to the result of the one-hot encoding vector, we can see the value of 1 in the second position, indicating that the pattern *x*^ω^ belongs to the second-class **B**.

To understand the explainability and the reason why the unknown pattern *x*^ω^ was classified as class **B**. Considering the unknown pattern *x*^ω^ after RBC conversion is: {110010}. Table 4 illustrates the samples and features that influenced the decision of the n-SBC model.

**Table 4:** Example of explicability of n-SBC.

**Pattern number**	**Class**	**Patterns in Train b^L^**	**Hamming difference vector against b^ω^**	**Value of Z^ω^**
*b* ^1^	A	101111	100010	2
*b* ^2^	A	111110	110011	4
*b* ^3^	B	010001	011100	3
*b* ^4^	B	000010	001111	4
*b* ^5^	B	110000	111101	5

Since *n* = 2, the two closest samples from each class are selected, which means that *b*^ω^ is classified as class B because they are very similar to patterns *b*^4^, and *b*^5^, which belong to class B. Moreover, since in the string *b*^ω^ and *b*_*i*_, in this case, each feature of the dataset is represented by 3 bits of the vector; it can be observed that the pattern *b*^ω^ is similar to *b*^ω^ because it matches with the second feature, and it is similar to *b*^5^ because it matches the first feature in totality. In this way, we can understand why the model decided to classify this pattern into its corresponding class.

## 4 Results and discussion

In this part, we present the detailed analysis of the experimental stage of our proposed algorithm against well-known state-of-the-art classification models. Subsection 4.1 describes the dataset selected in the experimental stage. Subsection 4.2 explains the validation method used, while 4.3 describes the performance measures. Subsection 4.4 shows the results obtained using the experimental methods and metrics described, and subsection 4.5 discusses the statistical significance results comparison.

### 4.1 Datasets

For the experimental phase of the present work, 20 datasets were selected, each representing a variety of diseases, with a focus on chronic conditions.

These data sets were mainly obtained from three widely known repositories: the KEEL repository (available at https://sci2s.ugr.es/keel/index.php), the UCI Machine Learning repository (accessible at https://archive.ics.uci.edu/ml/index.php), and the Kaggle repository (found at https://www.kaggle.com/datasets). To facilitate a deeper understanding, a complete description of each selected data set has been compiled. This compilation is summarized in Table 5, which provides information on the dataset's features, including the nature of the diseases it represents, the data structure, and the class imbalance index.

**Table 5:** Datasets description.

**Datasets**	**Features**		**Patterns**	**IR**	**Classes**
	**Categorical**	**Numerical**			
Appendicitis	0	7	106	4.04	2
Exasens COPD	0	7	80	1.00	2
Acute Inflammations D1	5	1	120	1.03	2
Acute Inflammations D2	5	1	120	1.40	2
ACPs Lung Cancer	38	0	901	31.25	4
Vertical Column 2C	0	6	310	2.1	2
Contraceptive	5	4	1,473	1.88	3
Cryotherapy	0	6	90	1.14	2
Dermatology	1	33	366	5.6	6
Hepatitis	12	7	155	3.84	2
Mammographic Masses	0	5	961	1.15	2
Wisconsin	0	9	683	1.85	2
HCC Survival	0	50	165	1.61	2
Autism Adolescent	8	12	104	1.29	2
Autism Child	8	12	292	1.07	2
Survey Lung Cancer	14	1	309	6.90	2
Breast Cancer Coimbra	0	9	116	1.23	2
Saheart	1	8	462	1.88	2
Cirrhosis	0	44	267	3.85	3
Multiple Sclerosis	16	4	273	1.18	2

The imbalance index for each dataset was calculated as follows:


$$\begin{array}{l} IR=\frac{number\;of\;minority\;class\;patterns}{number\;of\;majority\;class\;patterns} \end{array}$$ (20)

In the following, we provide a brief description of the selected datasets.

**Appendicitis**: This dataset was collected at https://www.kaggle.com/datasets/timrie/appendicitis from the Kaggle repository. The dataset comprises seven medical measures for 106 patients, with classes indicating whether each patient has appendicitis. Kaggle Snapshot: appendicitis/timrie, downloaded 2024-09-18.

**Exasens COPD**: This data set aims (based on demographic information from saliva) to classify patients into four classes according to their membership: chronic obstructive pulmonary disease (COPD), asthma, respiratory infections, and completely healthy patients. The dataset was collected from the UCI Machine Learning Repository at https://archive.ics.uci.edu/ml/datasets/Exasens. Downloaded 2024-06-14.

**Acute Inflammations D1** and **Acute Inflammations D2**: These datasets are from a study aimed at detecting two urinary system diseases. Both datasets were obtained from the UCI Machine Learning repository at https://archive.ics.uci.edu/ml/datasets/Acute+Inflammations. Downloaded 2024-03-14.

**ACPs Lung Cancer**: This dataset was obtained from the UCI repository at https://archive.ics.uci.edu/ml/datasets/Anticancer+peptides, which contains information on peptides (amino acid codes) and their anticancer activity in lung cancer cell lines. Downloaded 2024-03-14.

**Vertical Column**: This dataset aims to detect if a patient has some vertebral column disease. It was recovered from the UCI Machine Learning repository at http://archive.ics.uci.edu/ml/datasets/vertebral+column. In vertebral Column 2C, the classes Disk Hernia and Spondylolisthesis were merged into a single class, labeled Abnormal. Downloaded 2024-03-14.

**Contraceptive**: This dataset was collected from the UCI Machine Learning repository at http://archive.ics.uci.edu/dataset/30/contraceptive+method+choice. It is used to predict the current contraceptive method from demographic and socioeconomic information. Downloaded 2024-03-14.

**Cryotherapy**: This dataset was collected from the UCI Machine Learning repository at https://archive.ics.uci.edu/dataset/429/cryotherapy+dataset, which contains treatment outcomes for 90 patients who underwent cryotherapy. It has two classes: successful and unsuccessful. Downloaded 2024-03-14.

**Dermatology**: This dataset was obtained from the UCI Machine Learning repository at https://archive.ics.uci.edu/dataset/33/dermatology, whose main aim is to determine the type of Eryhemato-Squamous Disease based on 34 patient attributes. Downloaded 2024-03-14.

**Hepatitis**: This dataset aims to detect hepatitis using simple tabular data from patients, most of whom have categorical data. Furthermore, the dataset has two classes and was collected from the UCI Machine Learning repository at http://archive.ics.uci.edu/dataset/46/hepatitis. Downloaded 2024-02-03.

**Mammographic Masses**: This dataset aims to distinguish between benign and malignant mammographic masses using BI-RADS attributes and patient age. The dataset was collected from https://archive.ics.uci.edu/dataset/161/mammographic+mass, in the UCI Machine Learning repository. Downloaded 2024-01-21.

**Wisconsin**: This dataset was collected from the UCI Machine Learning repository at https://archive.ics.uci.edu/ml/datasets/breast+cancer+wisconsin+(diagnostic), which describes cases from a study conducted at the University of Wisconsin Hospitals in Madison involving patients who had undergone surgery for breast cancer. The classification task is to determine if the detected tumor is benign or malignant. Downloaded 2024-02-24.

**HCC Survival**: This dataset was obtained from https://archive.ics.uci.edu/dataset/423/hcc+survival, in the UCI Machine Learning repository. It contains real clinical data from 165 patients diagnosed with HCC, with the aim of predicting 1-year survival after diagnosis. Downloaded 2024-03-28.

**Autism adolescent and Child**: These datasets were collected from the UCI Machine Learning repository at https://archive.ics.uci.edu/dataset/420/autistic+spectrum+disorder+screening+data+for+adolescent and https://archive.ics.uci.edu/dataset/419/autistic+spectrum+disorder+screening+data+for+children, respectively. The idea of both datasets is to detect Autistic Spectrum Disorder. Downloaded 2024-04-02.

**Survey Lung Cancer**: The classification task in this dataset is to determine whether a given patient has lung cancer, based on variables collected via a survey. The set was obtained from the Kaggle repository at https://www.kaggle.com/mysarahmadbhat/lung-cancer. Kaggle Snapshot: Lung Cancer/Mysar Ahmad Bhat, downloaded 2024-04-13.

**Breast Cancer Coimbra**: This dataset was collected from https://archive.ics.uci.edu/dataset/451/breast+cancer+coimbra, in the UCI Machine Learning Repository. The dataset comprises clinical features from 64 patients. Downloaded 2024-02-22.

**Saheart**: This dataset aims to detect patients with heart diseases but was built for Stanford University and was collected at https://web.stanford.edu/~hastie/ElemStatLearn//datasets/SAheart.data. Downloaded 2024-02-24.

**Cirrhosis**: This dataset comprises 17 clinical features for predicting patient survival in patients with liver cirrhosis, collected from the UCI Machine Learning repository at https://archive.ics.uci.edu/dataset/878/cirrhosis+patient+survival+prediction+dataset-1. Downloaded 2024-02-22.

**Multiple Sclerosis**: The classification task in this dataset is to detect multiple sclerosis using patient information, such as personal data, symptoms, and metrics from medical tests. The dataset was collected from https://www.kaggle.com/datasets/desalegngeb/conversion-predictors-of-cis-to-multiple-sclerosis/data, the Kaggle repository. Kaggle Snapshot: Multiple Sclerosis Disease/A Legacy Grandmaster!, downloaded 2024-04-11.

### 4.2 Validation methods

In this section, we describe the validation method used in the experimentation stage. To obtain reliable results when measuring classifier performance during the experimentation stage, it is necessary to have previously implemented a validation method that divides the original dataset into two sets: a test set and a learning set.

**Figure 1:**
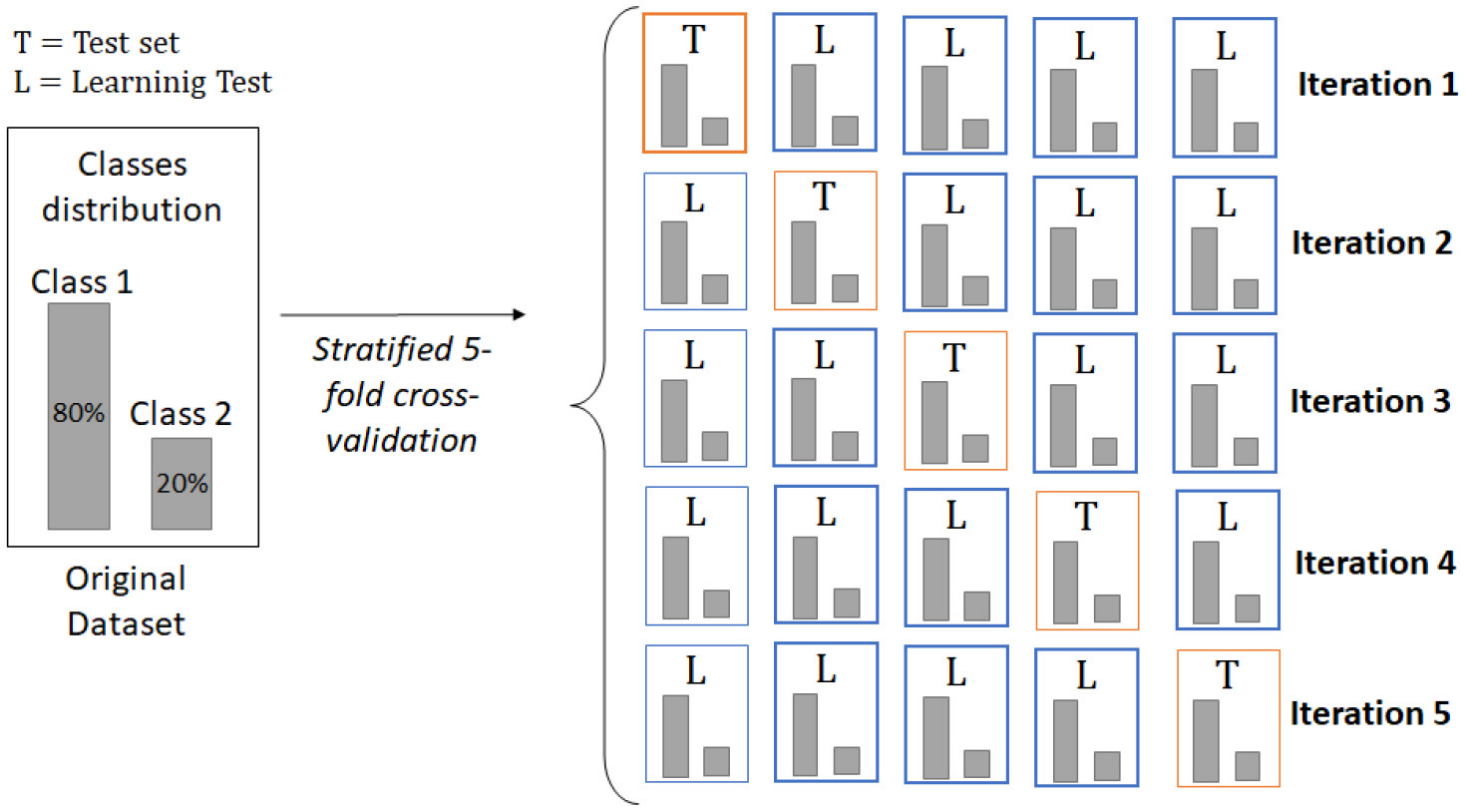
Stratified five-fold cross-validation method.

One of the most widely used methods is *k-*fold cross-validation, which randomly divides the original set into k equal-sized subsets (folds), using one fold as the test set and the rest as the training set. This process is repeated k times in order to use all folds at least once as test sets (Wong, 2015; Sarker, 2021). On the other hand, there is a stratified version of this validation method, called stratified *k-*fold cross-validation, which is highly recommended for data sets with class imbalance, since it attempts to preserve approximate class proportions within each fold. In this way, the test sets created in each iteration present as much as possible the class distribution of the original set, which helped mitigate errors caused by class bias (Derrac et al., 2015; Nakatsu, 2020). Figure 1 shows the operations of the stratified *k-*fold cross-validation when *k* = 5.

Given the class-imbalanced datasets used in the current study, stratified *k-*fold cross-validation with *k* = 10 has been employed to maintain approximately equal proportions of patterns per class across folds.

### 4.3 Performance measures

**Figure 2:**
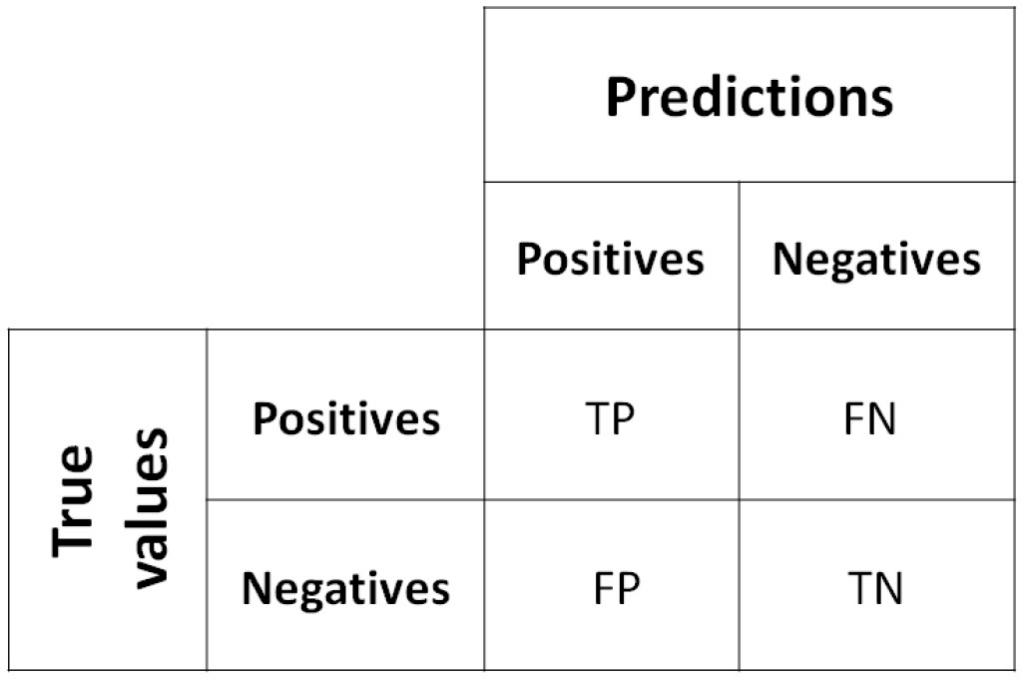
Confusion matrix for a bi-class dataset.

The evaluation of classifier performance is a crucial area of interest in specialized literature. The most popular and naturally simple way to measure performance is to use the accuracy metric, which calculates the percentage of patterns in the test set that are correctly classified; that is, it counts the total number of correctly classified patterns with respect to the total number of patterns. However, there is a way to more completely represent the results of the classifier's performance, which is called a confusion matrix, as shown in Figure 2, which consists of four possible cases within a two-class classification problem (García et al., 2010a), where each cell in the confusion matrix represents TP (true positive), TN (true negative), FP (false positive), and FN (false negative).

As mentioned above, one of the most popular metrics for measuring classifier performance is accuracy. In the case of bi-class problems, and using the confusion matrix as a basis, the metric can be expressed as in the equation:


$$\begin{array}{l} Accuracy=\frac{TP+TN}{TP+FN+FP+TN} \end{array}$$ (21)

However, more robust metrics have emerged in the literature to mitigate the limitations of the accuracy metric, which is not suitable for class-imbalanced datasets, a common data complexity mainly found in medical datasets. This data complexity harms the evaluation of the classifier's performance, yielding metrics that do not truly reflect the algorithm's capacity (López et al., 2013).

First, the sensitivity metric will be described, which measures the probability that the classifier returns a positive result when the instance is a true positive. The sensitivity metric can be expressed as follows (García et al., 2010b).


$$\begin{array}{l} Sensitivity=\frac{TP}{TP+FN} \end{array}$$ (22)

On the other hand, there is another crucial metric, the counterpart of the sensitivity metric: the specificity metric. This metric estimates the probability that the classifier will return a negative result when the instance is actually negative (García et al., 2010b).


$$\begin{array}{l} Specificity=\frac{TN}{TN+FP} \end{array}$$ (23)

There are different metrics for different purposes, such as the area under the ROC curve (AUC), precision, F1 score, and balanced accuracy (BA), but the majority of them are calculated from the confusion matrix (García et al., 2010b). Because the datasets selected for this study exhibit class imbalance, it was decided to use the Balanced Accuracy (BA) performance metric, which is recommended for such cases (López et al., 2013; García et al., 2010b). The BA metric is calculated from the performance metrics Sensitivity and Specificity, which represent the average of both measures.


$$\begin{array}{l} BA=\frac{Sensitivity+Specificity}{2} \end{array}$$ (24)

On the other hand, the value of BA in multi-class datasets, for k classes, is calculated as follows:


$$\begin{array}{l} BA=\frac{1}{k}\overset{k}{\underset{i=1}{\sum }}\frac{T_{i}}{N_{i}}, \end{array}$$ (25)

where *T*_*i*_ is the number of patterns correctly classified in class *i*, and *N*_*i*_ represents the total number of patterns within the dataset of class *i*.

**Figure 3:**
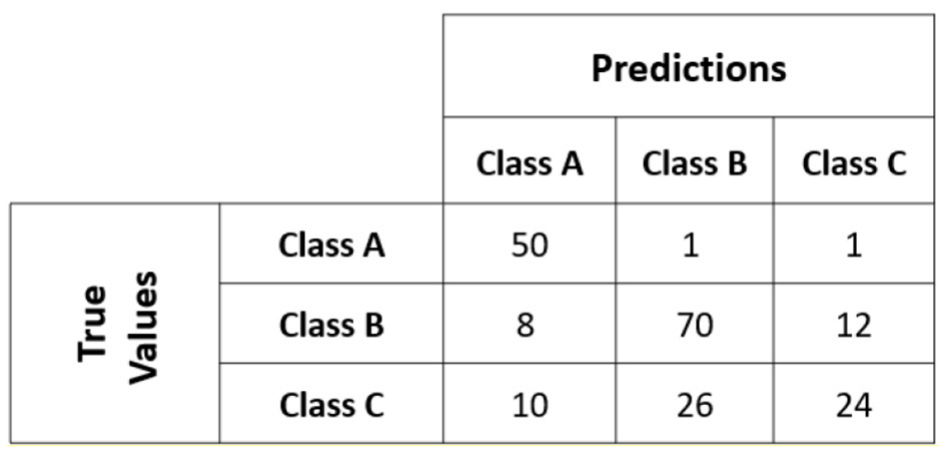
Example of a confusion matrix for a multi-class dataset.

Example. Figure 3 shows a confusion matrix for an unbalanced dataset, with 170 patterns in class A and 30 in class B. Therefore, the similar dataset has a very severe class imbalance; its imbalance index is IR = 5.6.

For class *i* ∈ {*A, B, C*} sensitivity (*T*_*i*_/*N*):


$$\begin{array}{l} Sensitivity_{A}=\frac{TP_{A}}{TP_{A}+FN_{A}}=\frac{50}{50\;+\;1+1}=0.96 \end{array}$$ (26)

$$\begin{array}{l} Sensitivity_{B}=\frac{TP_{B}}{TP_{B}+FN_{B}}=\frac{70}{70\;+\;8+12}=0.77 \end{array}$$ (27)

$$\begin{array}{l} Sensitivity_{C}=\frac{TP_{C}}{TP_{C}+FN_{C}}=\frac{24}{24\;+\;10\;+\;26}=0.40 \end{array}$$ (28)

In this example, the Balance Accuracy (BA) value of the confusion matrix is as follows:


$$\begin{array}{l} BA=\frac{1}{3}\left(0.96\;+\;0.77\;+\;0.40\right)=0.713 \end{array}$$ (29)

### 4.4 Time complexity analysis

Table 6 compares the time complexities of the classification algorithms used in the present study and of the proposed n-SBC model.

**Table 6:** Comparison of time complexities between algorithms.

**Algorithm**	**Spatial**	**Time**	
		**Training**	**Inference**
n-SBC	*O*(|*L*|*B)	*O*(|*L*|*B)^a^	*O*(|*L*|*B)
k-NN	*O*(|*L*|*B)	*O*(1)	*O*(|*L*|*B)
SMO	*O*(*n*_sv_*B)	*O*(|*L*|^2^*B)	*O*(*n*_sv_*B)
Naïve Bayes	*O*(C*B)	*O*(|*L*|*B)	*O*(C*B)
C4.5	*O*(|*L*|)	*O*(|*L*|*B*log|*L*|)	*O*(log|*L*|)
Random Forest	*O*(T*|*L*|)	*O*(T*|*L*|*B*log|*L*|)	*O*(T*log|*L*|)
MLP	*O*(B*H + H*C)	*O*(I*|*L*|*(B*H + H*C))	*O*(B*H + H*C)

^a^Time complexity for converting all dataset features to their RBC binary strings and concatenating them to create the memory *M* (preparing the training process in the n-SBC classifier).

**Notation**. **n**_**sv**_: Number of support vectors in SVM; **T:** Number of trees in Random Forest; **H:** Number of hidden units in MLP; **I:** Number of epochs in MLP; |**L**|**:** Total number of patterns (instances) in the training dataset; **C:** Number of classes in the data set; **X**^ω^**:** Unknown pattern (test) to be classified; **d:** number of features; **b**_**i**_**:** RBC bit-length of feature *i*; $B=\overset{d}{\underset{i=1}{\sum }}b_{i}$ (total bits per encoded pattern); **B:** Length of each pattern in the binary string generated by the RBC encoder.

### 4.5 Classification results

Table 7 compares the performance of the proposed algorithm with that of different classifiers across the 20 datasets described earlier. The algorithms used for comparison were run in *Weka version 3.8.2*, using the tool's default hyperparameters. The results of the n-SBC algorithm were obtained using *MATLAB R2021b* with a random seed of 1. For the experimental process, we evaluated two pre-specified SBC variants with *n* ∈ {3, 5}. These values were pre-selected once from a preliminary sweep *n* ∈ {1, 2, 3, 4, 5} using training-only validation and were then held fixed across all datasets. To ensure a fair comparison, the study does not cherry-pick the best *n* values in the classification results; instead, those are explicitly excluded from the Friedman and Holm statistical tests to avoid inflating the number of comparisons.

**Table 7:** Results of the balanced accuracy measurement obtained by the classifiers.

**Dataset**	**Naïve Bayes**	**IB1**	**IB3**	**MLP**	**SMO**	**C4.5**	**Random Forest**	**3-SBC**	**5-SBC**
Appendicitis	**0.786**	0.745	0.738	0.75	0.744	0.732	0.744	0.703	0.746
Exasens_copd	0.9	0.937	0.875	**0.95**	0.887	0.887	0.912	0.898	0.9
Acute inflammation d1	0.992	**1**	**1**	**1**	**1**	**1**	0.933	**1**	**1**
Acute inflammation d2	**1**	**1**	**1**	**1**	**1**	**1**	0.958	**1**	**1**
ACPs lung cancer	0.695	0.683	0.648	0.707	0.707	0.559	0.645	**0.984**	**0.984**
Column 2c	0.801	0.809	0.751	0.807	0.704	0.77	**0.82**	0.712	0.735
Contraceptive	0.514	0.417	0.42	0.54	0.488	0.488	0.507	**0.637**	0.636
Cryotherapy	0.841	0.9	0.911	0.879	0.879	0.936	0.936	0.934	**0.941**
Dermatology	**0.976**	0.952	0.969	0.968	0.971	0.955	0.958	0.967	0.971
Hepatitis	0.83	0.736	0.763	0.755	0.807	**0.85**	0.835	0.818	0.835
Mammographic Masses	0.828	0.754	0.763	0.822	0.796	0.822	0.797	**0.84**	0.834
Wisconsin	0.964	0.94	0.964	0.939	**0.965**	0.937	0.963	0.939	0.941
HCC Survival	0.677	0.6	0.584	0.6	0.711	0.546	0.668	**0.828**	0.822
Autism Adolescent	0.959	0.882	0.841	0.887	0.891	**1**	**1**	0.923	0.902
Autism Child	0.827	0.748	0.784	0.798	0.829	0.819	0.812	0.969	**0.976**
Survey lung cancer	0.688	0.758	0.745	**0.802**	0.782	0.747	0.754	0.792	0.765
Breast Cancer Coimbra	0.63	0.67	0.674	0.651	0.663	0.688	0.735	**1**	**1**
Saheart	0.655	0.58	0.619	0.63	**0.658**	0.657	0.622	0.577	0.57
Cirrhosis	0.515	0.429	0.444	0.525	0.52	0.549	0.524	**0.642**	0.617
Multiple Sclerosis	0.902	0.788	0.802	0.907	0.812	0.783	0.786	**0.984**	0.983
**Times Best BA**	3	2	2	4	4	4	2	**9**	**7**

They were used with the class-imbalance complexity specified in Table 5 across all classifiers, and the results were compared in Table 7. The results that achieve competitive performance relative to the other classifiers for each dataset are highlighted in bold.

No preprocessing other than handling missing values and converting categorical values to numeric was applied, as explained in Section 3. No samples were removed from the datasets, nor were synthetic samples added; they also kept the original sizes and format.

The proposed algorithm achieved competitive performance across nine of the twenty-one datasets. For example, it performed well on Acute Inflammation d1 and d2, ACP lung cancer, contraceptive use, mammographic masses, HCC survival, breast cancer Coimbra, cirrhosis, and multiple sclerosis.

Furthermore, Table 7 shows some cases where the classifiers achieved 1 on the balanced accuracy metric. This indicates that it was perfect, i.e., the classifier made zero errors. Thus, if we count the frequency at which classifiers obtained these cases, our proposed model was one of the highest-performing BA in both versions (3-SBC and 5-SBC), receiving it in 3 out of 21 datasets.

Similarly, the algorithm that performed best across the majority of datasets was our proposed 3-SBC model, which was the best in 9 of 20 datasets, followed by our other model, 5-SBC, which was the best in 7 of 20 datasets.

Nevertheless, datasets with high data complexity that obtained inadequate scores were Cirrhosis, Saheart, HCC Survival, and Contraceptive, among which our proposed models achieved the highest performance in 3 out of 5 cases. This happens due to the No Free Lunch theorem; therefore, it is expected that our proposed models will not be the best-performing classifiers across all datasets. This theorem indicates that no classifier is capable of being the best on all types of problems (Wolpert and Macready, 1997; Adam et al., 2019). Furthermore, the classifier with the best performance also performed poorly, such as Saheart, which achieved 0.658 on SMO.

However, in favor of our proposal, it can be noted that, in most cases, the performances of the 3-SBC classifier do not vary overly from the high balanced accuracy values obtained by other classifiers; such is the case of the Survey lung cancer, Dermatology, and Wisconsin datasets in which our proposed model 3-SBC obtained very similar results against the best models in those cases, such as SMO or Naïve Bayes.

### 4.6 Statistical analysis

Comparing various machine learning algorithms and selecting a final model or algorithm as the winner is a common practice in machine learning, model research, and applications. Models in relation to a set of experiments are evaluated using a validation method, e.g., *k-*fold cross-validation or leave-one-out cross-validation (a particular case of *k-*fold cross-validation where k equals the number of instances in the dataset), and the results are directly compared by calculating a performance measure. While this is a simple and somewhat intuitive approach, it is difficult to determine whether a difference is due to the algorithm's real capability or a statistical fluke.

It is crucial to distinguish genuine performance differences from statistical flukes. Therefore, it is necessary to apply statistical hypothesis testing, which addresses this issue by quantifying the probability of observing score differences under the null hypothesis that scores are drawn from the same distribution. Rejection of this null hypothesis indicates that the observed differences are statistically significant, rather than due to chance.

In this context, to conduct a more reliable comparative analysis, it was proposed to use Friedman's test (Friedman, 1937) to determine whether there are significant differences in the yields observed during the experiment.

Table 8 shows the performance obtained by the different classification algorithms proposed. After performing Friedman's statistical test, the null hypothesis was rejected at the 95% confidence level (*p*-value = 0.000516), indicating statistically significant differences among the classifiers.

**Table 8:** Friedman's means ranks table.

**Algorithm**	**Mean ranks^a^**
5-SBC	3.2143
3-SBC	3.7857
Naïve Bayes	4.5952
MLP	4.8095
SVM	4.881
Random Forest	5.1429
C4.5	5.4762
1-NN	6.4762
3-NN	6.619

^a^sorted from best ranked to worst.

The proposed models (5-SBC and 3-SBC) rank first in the Friedman mean rank calculation concerning the remaining seven algorithms, while the *k-*NN family algorithms rank last in the Friedman mean rank table.

On the other hand, a *post-hoc* test, the Holm test (Holm, 1979), was applied. The results in Table 9 reject the hypothesis at an adjusted *p*-value of ≤ 0.05. Therefore, significant performance differences between the two versions of the proposed algorithm and the remaining state-of-the-art algorithms used in the study are demonstrated. In particular, it can be observed that, considering the best algorithm according to the Friedman test, the 5-SBC algorithm, it has significant differences above the 95% confidence level for the 1-NN and 3-NN algorithms; on the other hand, the SVM, MLP, and Naïve Bayes algorithms obtained *p*-values (although higher than the corrected threshold) that indicate a possible significant difference to the 5-SBC model, which could be interpreted as marginal evidence in exploratory contexts.

**Table 9:** Results obtained by Holm's *post-hoc* test.

***i***.	**Algorithm**	**z** = (**R_0_−R_i_**)**/SE**	***p***	**Holm**
8	3-NN	3.695042	0.000220	0.00625
7	1-NN	3.579572	0.000344	0.00714
6	C4.5	2.540341	0.011074	0.00833
5	Random Forest	2.078461	0.037667	0.01000
4	SVM	1.876388	0.060602	0.01250
3	MLP	1.616581	0.105969	0.01666
2	Naïve Bayes	1.587713	0.112351	0.02500
1	3-SBC	0.692820	0.488422	0.05000

After presenting the experiments, it was observed that the proposed algorithm obtained competitive results. This conclusion is supported by statistically significant differences in the n-SBC algorithm's observed performance across two of the seven selected classifiers on the same set of classification datasets.

Consequently, the results corroborate the hypothesis that the proposed n-SBC algorithm is indeed competitive for classification and disease prediction, as the majority of the datasets used focus on detecting different diseases.

## 5 Conclusion and future research

In this research work, a new proposed model, n-SBC (n Similarity Binary Classifier), was presented, along with an experimental analysis to verify its effectiveness against other state-of-the-art algorithms on datasets related to medicine.

Similarly, the advantages of the proposed model were described, as were its simplicity, explainability, and its ability to address imbalance, a data complexity that is very common in the literature.

The detailed results in this research, presented in Section 4, highlight the capacity of the proposed algorithm, specifically the version 5-SBC, due to its competitive performance compared to other popular classification algorithms in the literature. Similarly, this research explores and presents in detail a new approach that uses the similarity between binary strings as the basis for a machine learning model while maintaining simplicity and effectiveness. Above all, the proposed novel algorithm promotes the research and application of explainable AI, which is of great contribution to specific areas, such as health or finance.

The proposed model has a limitation in handling pattern cardinality, as it converts patterns to binary strings, which can increase computational complexity during classification. Therefore, as future research, it is proposed to develop a method or pursue a completely new approach that can solve the problem identified in the proposed model while maintaining its simplicity, explainability, and performance. It is under consideration for implementation and demonstrates a novel similarity measure that can improve the model's performance while maintaining the algorithm's simplicity and explainability. On the other hand, an important objective is to apply the proposed n-SBC model to image classification tasks, specifically to medical images (x-ray mainly), due to its ease in preprocessing to adapt it and be able to use the n-SBC model with evolutionary algorithms or metaheuristic processes, with the goal of optimizing the model's performance. Another aspect to consider in future studies is analyzing the model's behavior on datasets with outliers to evaluate its robustness and adaptability to more complex, noisy scenarios. Finally, we plan to extend n-SBC by incorporating the three-way decision (3WD) rule based on model margin to explicitly handle classification uncertainty, compare granular 3WD variants, and report risk-coverage improvements on medical data.

## Data Availability

Publicly available datasets were analyzed in this study. This data can be found here: https://archive.ics.uci.edu/datasets.
