# Supplementary material for: Medical pattern classification using a novel binary similarity approach based on an associative classifier
Source: Front Artif Intell. 2026 Jan 14;8:1610856. doi: 10.3389/frai.2025.1610856 (PMC12847284; doi:10.3389/frai.2025.1610856)
Supplement: Supplementary file 1 [file Data_Sheet_1.pdf]

Supplementary Material

Medical Pattern Classification Using a Novel Binary Similarity Approach based on an Associative Classifier

Osvaldo Velazquez-Gonzalez [1*], Cornelio Yañez-Marquez[1*], Antonio Alarcón-Paredes[1*]

[1]Centro de Investigación en Computación, Instituto Politécnico Nacional, CDMX, México;

*** Correspondence:**
Cornelio Yanez-Marquez
cyanez@cic.ipn.mx

1 Supplementary Data

1.1 Additional Classification Results

In table 7, it shows some cases where the n-SBC algorithm or other algorithms achieve perfect classification. First, Breast Cancer Coimbra dataset performed perfectly BA = 1 only on both variations of n-SBC. This dataset has 116 patterns, with zero duplicates, 9 numerical features and 0 categorical, it has no class imbalance, since it has an IR of 1.23 with only two classes. Random Forest was the best performing baseline model, with 0.735, using the validation method used in this work, 10-fold stratified cross validation, the confusion matrix is:

	Predicted Positive	Predicted Negative
True Positive	35	17
True Negative	13	51

$$Sensitivity = 0.673; Specificity = 0.797$$

$$Precision = 0.750; f1\ score = 0.772$$

With the aim of ruling out that the dataset has leaked labels in the features, the feature importance matrix was calculated and extracted using the Random Forest model (in Weka 3.8.2), which indicated that the 3 most important features for the classification are the features: BMI (numerical) with a value of 0.46, age (numerical) with a value of 0.44 and Glucose (numerical) with a value of 0.37. Finally, after a correlation matrix analysis, the most correlated features are insulin ↔ HOMA with a value $r = 0.932$, which indicates a strong positive linear relationship; glucose ↔ HOMA with $r = 0.696$, and Age ↔ leptin with $r = 0.57$.

Using both variants of the n-SBC, the confusion matrix is:

	Predicted Positive	Predicted Negative
True Positive	52	0
True Negative	0	64

Therefore, it could be concluded that the variants of the n-SBC models were able to completely differentiate between the two classes. On the other hand, the datasets Acute inflammations d1 and d2 have 120 patterns, with 21 duplicate rows, 5 categorical features and only one numerical. The version d1 has 1.03 of IR while version d2 has 1.40 of IR. First, with dataset version d1 and using Random Forest as demonstration since it was the only classifier that did not obtain $BA = 1$, the confusion matrix is:

	Predicted Positive	Predicted Negative
True Positive	55	6
True Negative	2	57

$$Sensitivity = 0.966; Specificity = 0.902$$

$$Precision = 0.904; f1\ score = 0.934$$

Using both variants of the n-SBC, the confusion matrix is:

	Predicted Positive	Predicted Negative
True Positive	61	0
True Negative	0	59

The feature importance was calculated and extracted using the Random Forest model (in Weka 3.8.2), which indicated that the 3 most important features for the classification in the Acute inflammations d1 are the features: urine-pushing with a value of 0.83, lumbar-pain with a value of 0.81 and nausea with a value of 0.71.

Finally, using the dataset acute inflammation d2, Random Forest obtained the following confusion matrix:

	Predicted Positive	Predicted Negative
True Positive	65	5
True Negative	0	50

$$Sensitivity = 1.0; Specificity = 0.928$$

$$Precision = 0.909; f1\ score = 0.52$$

The feature importance was calculated and extracted using the Random Forest model (in Weka 3.8.2), which indicated that the 3 most important features for the classification in the Acute inflammations d2 are the features: temperature with a value of 0.83, lumbar pain with a value of 0.77, and nausea with a value of 0.74. The n-SBC variants obtain perfect performance and confusion matrix as Acute Inflammations d1.

On the other hand, after removing all duplicates from Acute Inflammations d1 and d2, these are the results obtained by the classifiers.

Table 1. Additional results from dataset without duplicates samples.

Dataset	Naïve Bayes	IB1	IB3	MLP	SMO	C4.5	Random Forest	3-SBC	5-SBC
Acute inflamations d1	0.989	**1**	**1**	**1**	**1**	**1**	0.870	**1**	**1**
Acute inflamations d2	**1**	**1**	**1**	**1**	**1**	**1**	0.918	**1**	**1**

After rerunning the experiments for acute inflammations d1 and d2 without duplicate values, the results remain very similar to the original dataset results, suggesting that the perfect BA values are not due to data leak problems.

On the other hand, the results of all versions of n-SBC, when $n \in \{1,2,3,4,5\}$ in the 21 data sets are shown below.

Table 2. Results of the balanced accuracy measurement obtained by the n-SBC versions $\{1,2,3,4,5\}$.

Dataset	1-SBC	2-SBC	3-SBC	4-SBC	5-SBC
Appendicitis	0.724	0.727	0.703	0.725	**0.746**
Exasens_copd	0.775	0.847	0.898	**0.900**	**0.900**
Acute inflamations d1	**1**	**1**	**1**	**1**	**1**
Acute inflamations d2	**1**	**1**	**1**	**1**	**1**
ACPs lung cancer	0.983	0.983	**0.984**	0.983	**0.984**
Column 2c	0.661	0.680	0.712	0.720	**0.735**
Contraceptive	**0.652**	0.645	0.637	0.636	0.636
Cryotherapy	0.932	**0.943**	0.934	0.922	0.941
Dermatology	0.963	0.956	0.967	0.966	**0.971**
Hepatitis	0.793	0.817	0.818	0.818	**0.835**
Mammographic Masses	**0.847**	0.841	0.840	**0.836**	0.834
Wisconsin	**0.944**	0.941	0.939	0.942	0.941
HCC Survival	0.810	0.824	**0.828**	0.827	0.822
Autism Adolescent	0.902	**0.923**	**0.923**	0.902	0.902
Autism Child	0.957	0.966	0.969	**0.976**	**0.976**
Survey lung cancer	0.777	**0.792**	**0.792**	0.778	0.765
Breast Cancer Coimbra	**1**	**1**	**1**	**1**	**1**
Saheart	**0.593**	0.586	0.577	0.567	0.570
Cirrhosis	**0.648**	0.636	0.642	0.618	0.617
Multiple Sclerosis	**0.991**	0.983	0.984	0.984	0.983
**Times Best BA**	9	6	7	6	**10**

As can be seen in Table 2, the 5-SBC version of the n-SBC model performed best, followed by the 1-SBC version. However, it is observed that, in all the n-SBC versions analyzed, the BA values remained consistent and very similar.
